# Methylated PIH1D1 as a Heart-Specific Biomarker for Anthracycline-Induced Cardiac Remodeling in Breast Cancer Patients

**DOI:** 10.1016/j.jacbts.2026.101510

**Published:** 2026-03-11

**Authors:** Po-Yen Hsu, Wan-Hong Huang, Yi-Yun Lee, Robert Passier, Szu-Chin Li, Chong-Lin Hong, Shih-Kai Hung, Hong-Yi Lin, Chun-Hung Lin, Chen-Yu Chien, Yi-Da Li, Hsiang-Chun Lee, Laurent Désaubry, Canan G. Nebigil, Michael W.Y. Chan

**Affiliations:** a Department of Biomedical Sciences, National Chung Cheng University, Chiayi, Taiwan; b Epigenomics and Human Diseases Research Center, National Chung Cheng University, Chiayi, Taiwan; c INSERM UMR 1260, Regenerative Nanomedicine, University of Strasbourg, FMTS (Fédération de Médecine Translationnelle de l'Université de Strasbourg), Strasbourg, France; d Applied Stem Cell Technologies, Department of BioEngineering Technologies, TechMed Centre, University of Twente, Twente, the Netherlands; e Division of Hematology and Oncology, Dalin Tzu Chi Hospital, The Buddhist Tzuchi Medical Foundation, Chiayi, Taiwan; f School of Medicine, Tzu Chi University, Hualian, Taiwan; g Department of Radiation Oncology, Dalin Tzu Chi Hospital, The Buddhist Tzuchi Medical Foundation, Chiayi, Taiwan; h Division of General Surgery, Dalin Tzu Chi Hospital, The Buddhist Tzuchi Medical Foundation, Chiayi, Taiwan; i Division of Cardiology, Dalin Tzu Chi Hospital, The Buddhist Tzuchi Medical Foundation, Chiayi, Taiwan; j Division of Cardiology, Department of Internal Medicine, Kaohsiung Medical University Gangshan Hospital, Kaohsiung, Taiwan; k Department of Internal Medicine, School of Medicine, College of Medicine, Kaohsiung Medical University, Kaohsiung, Taiwan; l Lipid Science and Aging Research Center, Kaohsiung Medical University, Kaohsiung, Taiwan; m Research Center for Precision Environmental Medicine, Kaohsiung Medical University, Kaohsiung, Taiwan

**Keywords:** breast cancer, cardiotoxicity, DNA methylation, doxorubicin, PIH1D1

## Abstract

Anthracyclines, key chemotherapy agents, pose cardiotoxicity risks. In a 3-year study of 89 breast cancer patients treated with doxorubicin or epirubicin, more than 50% showed reduced left ventricular ejection fraction and progressive ventricular dilation. Although troponin-I flagged acute damage, it failed to predict long-term remodeling. Using a human methylome atlas, researchers identified 33 heart-specific methylated CpG sites and validated methylated *PIH1D1* (m*PIH1D1*) as a novel biomarker. Elevated m*PIH1D1* levels strongly correlated with ventricular dilation but not left ventricular ejection fraction decline, indicating its sensitivity to early cardiac remodeling. m*PIH1D1* may complement troponin-I in risk assessment and cardiotoxicity management for patients undergoing anthracycline-based chemotherapy.

## Highlights

• Anthracycline therapy induces early ventricular dilation and a decline in LVEF, reflecting subclinical remodeling toward dilated cardiomyopathy.
• Circulating m*PIH1D1* serves as a heart-specific cfDNA biomarker for early cardiac injury detection.
• Elevated m*PIH1D1* levels at 3 to 6 months predict subsequent ventricular dilation and LVEF reduction with high diagnostic accuracy.
• Regimen analysis shows Lipo-DOX mitigates remodeling, whereas anthracycline + 5-FU aggravates dilation.
• Integrating cfDNA methylation with echocardiography enables precision monitoring and early cardioprotection in cardio-oncology.

## Visual Abstract

**Figure:**
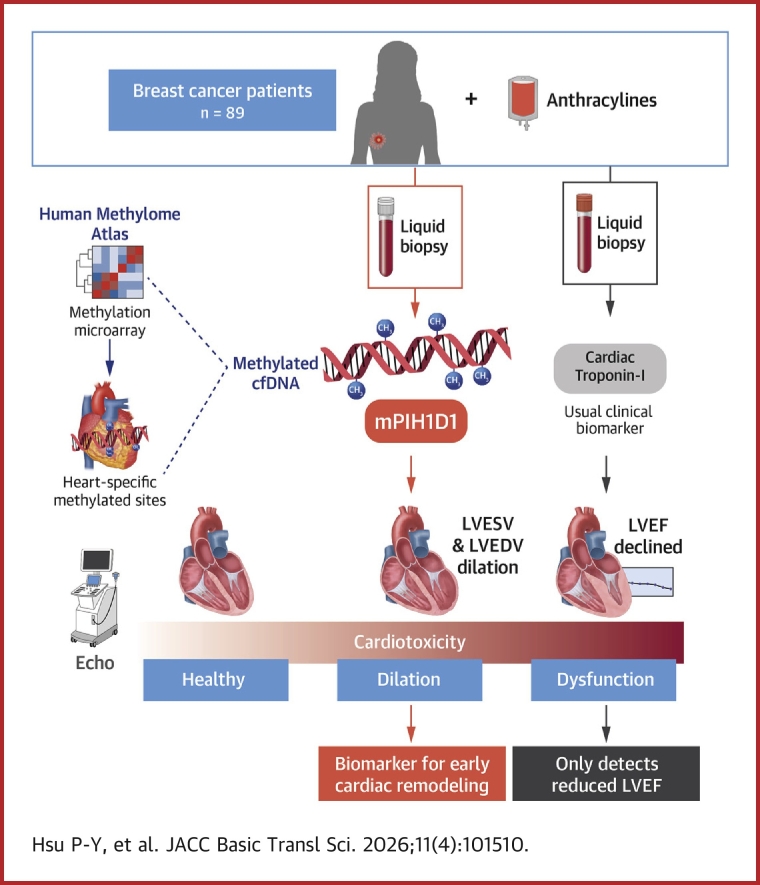

Anthracyclines are a mainstay in the treatment of various malignancies, including breast cancer, leukemia, and lymphoma. Although their efficacy in reducing tumor burden is well-documented, the clinical application of anthracyclines is markedly restricted by its potential to induce cardiotoxicity, which can lead to heart failure and other cardiovascular complications. Anthracycline-induced cardiotoxicity can cause dose-dependent damage to cardiomyocytes, resulting in alterations in cardiac function and structure.^1^^,^^2^ The underlying mechanisms of this toxicity are complex and multifactorial, involving oxidative stress, free radical formation, and mitochondrial dysfunction.^3^^,^^4^ As the incidence of cancer survivorship increases, understanding the underlying mechanisms, developing effective prevention strategies, and identifying reliable biomarkers for early detection of anthracycline-induced cardiotoxicity have become increasingly important for improving patient outcomes and quality of life.

In clinical practice, several biomarkers, including cardiac troponin-I, B-type natriuretic peptide (BNP), and creatine kinase, have been employed to detect anthracycline-induced cardiotoxicity.^5^ Among these, troponin-I, a cardiac-specific protein involved in myocardial contraction, is released into the bloodstream following cardiomyocyte injury. Its high sensitivity to cardiac damage makes it a valuable tool for diagnosing cardiac injury, including that caused by anthracyclines, and for providing prognostic insights into treatment outcomes.^6^^,^^7^ Although troponin-I is a sensitive marker for cardiac injury, there are still some limitations. One of the most significant disadvantages is its lack of specificity for cardiotoxicity, because elevated troponin-I levels may also result from noncardiac conditions such as renal failure, pulmonary embolism, sepsis, or even exercise.^6^^,^^8^^,^^9^ Furthermore, while troponin-I levels can reflect myocardial injury and help predict declines in left ventricular ejection fraction (LVEF), these changes often occur only after functional impairment has already developed.^10^ Therefore, the identification of novel, specific biomarkers capable of detecting early and subclinical anthracycline-induced cardiotoxicity remains a critical unmet need.

Epigenetic modifications, particularly DNA methylation, play a critical role in the regulation of gene expression and maintenance of cellular identity.^11^^,^^12^ DNA methylation predominantly occurs at cytosine residues within CpG dinucleotides and exhibits tissue-specific patterns, enabling the determination of the tissue of origin for circulating cell-free DNA (cfDNA).^13^ Previous studies,^14^, ^15^, ^16^ including our own work,^17^, ^18^, ^19^, ^20^ have demonstrated the potential of cfDNA methylation as a biomarker for detecting various human diseases.^21^ This tissue specificity makes cfDNA methylation a promising tool for identifying tissue damage and disease states.^13^^,^^22^ However, its application in the context of anthracycline-induced cardiotoxicity remains largely unexplored.

In this study, we aimed to identify a novel plasma methylation-based biomarker for the early detection of cardiotoxicity in breast cancer patients treated with anthracyclines. We prospectively followed a cohort of 89 patients who received doxorubicin (DOX) or epirubicin, with serial assessments of cardiac function and biomarker levels over a 3-year period. Building on prior evidence linking troponin-I elevation with subsequent declines in LVEF, we explored the potential of cfDNA methylation profiling to enhance early detection of cardiac injury. In particular, we investigated the methylation status of PIH1 domain containing 1 (*PIH1D1*) in plasma cfDNA as a candidate biomarker. This approach may offer a more specific and sensitive means of identifying anthracycline-induced cardiotoxicity before overt functional impairment occurs, addressing a critical unmet need in cardio-oncology.

## Methods

### Patient samples

A prospective cohort of 89 breast cancer patients treated with anthracyclines were recruited from Buddhist Dalin Tzu Chi Hospital, Chiayi, Taiwan. Patients were followed with echocardiographic data at baseline (before chemotherapy) until 36 months after the start of chemotherapy. All experiments involving human samples were conducted in accordance with the Helsinki Declaration of 1975, as revised in 2000. This study was also approved by the Institutional Review Board of the Buddhist Dalin Tzu Chi Hospital, Chia-Yi, Taiwan (approval number: B10802019). Written informed consent was obtained from all participants.

### Cell culture

MCF7 and MDA-MB-231 human breast cancer cell lines were cultured in Dulbecco’s Modified Eagle Medium (Gibco, Thermo Fisher Scientific, Inc) supplemented with 10% fetal bovine serum (Invitrogen, Thermo Fisher Scientific, Inc) and 50 U/mL penicillin-streptomycin (Invitrogen). The AC16 human cardiomyocyte cell line was maintained in Dulbecco’s Modified Eagle Medium/F12 (1:1) supplemented with 1% GlutaMAX (Invitrogen), 12.5% fetal bovine serum, and 1% penicillin-streptomycin. Human induced pluripotent stem cell-derived cardiomyocytes (iPSC-CMs) were cultured in RPMI 1640 (Gibco) supplemented with 1% GlutaMAX, 1% NEAA, and 1% B27 without insulin. The iPSC-CMs were treated with 3 μmol/L DOX (Sigma-Aldrich) for 96 hours. Genomic DNA was subsequently extracted for analysis. All cells were incubated at 37 °C in a humidified atmosphere containing 5% CO_2_.

### Extraction and bisulfite conversion of cfDNA

Whole blood (12 mL) was collected into three 10-mL K2-EDTA tubes (BD Biosciences) and processed within 2 hours to minimize cellular degradation. Samples were centrifuged at 550 *g* for 10 minutes at room temperature, and the plasma layer was carefully transferred to 1.5-mL microcentrifuge tubes, avoiding disturbance of the buffy coat. Plasma was subsequently centrifuged at 16,000 *g* for 10 minutes at room temperature to remove residual cellular debris. The clarified plasma was aliquoted and stored at −80 °C until further use.

cfDNA was extracted from 1 mL of plasma using the QIAamp Circulating Nucleic Acid Kit (Qiagen GmbH) according to the manufacturer’s instructions. Extracted cfDNA was bisulfite converted using the EZ DNA Methylation-Gold Kit (ZYMO Research), also following the manufacturer's protocol.

### Methylation-specific PCR and quantitative real-time methylation-specific PCR

Gel-based methylation-specific PCR (MSP) was performed using Platinum Taq DNA Polymerase (Invitrogen). In vitro methylated human DNA (ZYMO) was used as a positive control for methylation. Amplified products were separated on a 10% nondenaturing polyacrylamide gel, stained with ethidium bromide, and visualized under UV illumination. For the detection of m*PIH1D1* in patient plasma samples, quantitative real-time methylation-specific PCR (qMSP) was employed. Primers for the MSP and qMSP are listed in Supplemental Table S1. The amount of methylated DNA was quantified by determining the threshold cycle (Ct) for each sample against a standard curve generated by the cloned MSP fragment (m*PIH1D1*).

### Biomarker measurements

High-sensitivity cardiac troponin I (hs-cTnI) and BNP levels were measured using chemiluminescent microparticle immunoassays on the Abbott Architect platform (Abbott Laboratories). hs-cTnI was quantified using the Architect STAT High Sensitive Troponin-I assay, while BNP was measured using the Architect BNP assay. Both assays are designed to quantitatively determine their respective biomarkers in human plasma. All measurements were performed in accordance with the manufacturer's instructions.

### Echocardiography

Echocardiographic assessments were performed using a GE Vivid E95 ultrasound system (GE HealthCare) equipped with an M5Sc-D 1.5 to 4.6 MHz phased-array transducer. All measurements were obtained by 2 experienced cardiologists with the patient in the left lateral decubitus position, in accordance with the guidelines of the American Society of Echocardiography and the European Association of Cardiovascular Imaging.^23^^,^^24^ Two-dimensional, M-mode, Doppler, and tissue Doppler imaging (TDI) were utilized to enable comprehensive evaluation of cardiac structure and function. For the present analysis, only M-mode echocardiographic data were used to quantify left ventricular end-diastolic volume (LVEDV), left ventricular end-systolic volume (LVESV), and LVEF. LVEF was calculated using the standard volumetric formula: LVEF (%) = ([LVEDV − LVESV]/LVEDV) × 100. M-mode was selected over three-dimensional or more advanced imaging modalities to minimize patient discomfort, as the breast cancer patients were either recovering from surgery or experiencing treatment-related fatigue, making shorter and less demanding procedures preferable. In addition, left ventricular systolic velocity (LVS′) was measured using TDI at the lateral mitral annulus, serving as an index of longitudinal systolic function. Diastolic function was assessed via pulsed-wave Doppler of mitral inflow velocities (E and A waves) and TDI-derived mitral annular velocities (E′), with the E/E′ ratio calculated to estimate left ventricular filling pressures.

### Identification of heart-specific methylated CpG sites

A comprehensive human methylome reference atlas containing data from 25 different human tissues or cell types, published by Moss et al,^25^ was used to identify heart-specific methylated CpG sites (HSMCs). CpG sites with methylation levels below 10% in all noncardiac tissues were initially selected to ensure tissue specificity. From this subset, CpG sites with a methylation level difference >10% between the left atrium and the average methylation level across all other tissues were further filtered. This 2-step strategy yielded 33 CpG sites, which were designated as HSMCs.

### TCGA data analysis

The NCI GDC (Genomic Data Commons) and TCGA (The Cancer Genome Atlas) BRCA Methylation450K data sets were downloaded from UCSC Xena.^16^ The methylation levels of the CpG site in *PIH1D1* (cg02184280) were analyzed across solid tissue normal, primary tumor, and metastatic samples.

### Statistical analysis

All statistical analyses were conducted using GraphPad Prism version 8.0.2 (GraphPad Software, Inc) or Python version 3.13.1 (Python Software Foundation, 2024). Longitudinal data were analyzed using linear mixed-effects models to account for within-patient correlation. Time was treated as a categorical fixed effect with baseline as the reference, and patient ID was included as a random intercept. Post hoc baseline-referenced contrasts were used to compare each follow-up time point with baseline, with *P* values adjusted for multiple comparisons using the Holm method. For biomarker-based stratification analyses, group, time, and their interaction were included as fixed effects to evaluate differences in longitudinal trajectories between groups. This approach was chosen because follow-up data were unbalanced and included missing observations. Changes between groups (eg, ER+ vs ER−) were assessed using Mann-Whitney *U* tests. A *P* value <0.05 was considered statistically significant. Logistic regression analysis was used for the receiver-operating characteristic (ROC) area under the curve (AUC) analysis to determine the AUC, sensitivity, and specificity of m*PIH1D1* fold change at 3 months and both 3 and 6 months to identify patients who subsequently develop ≥1.5-fold increase in LVEDV and LVESV by either 3 or 6 months. Model results are presented with 95% CI. The logistic regression model generated predicted probabilities, which were used for ROC analysis. The optimal classification cutoff was determined using Youden’s index (sensitivity + specificity − 1), applied to either the biomarker value or the predicted probability as appropriate for each model.

## Results

### Anthracycline treatment resulted in a decrease in LVEF in breast cancer patients

**Figure 1:**
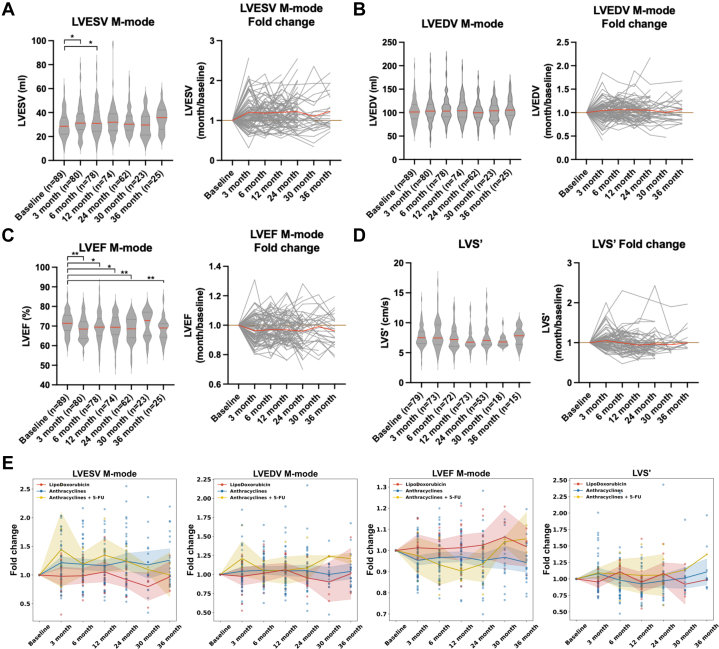
Doxorubicin Causes a Decrease in Ejection Fraction and Dilatation in Both Diastole and Systole (A) Left ventricular end-systolic volume (LVESV), (B) left ventricular end-diastolic volume (LVEDV), and (C) left ventricular ejection fraction (LVEF) were measured by M-mode echocardiography at baseline (pretreatment) and various post-treatment (3, 6, 12, 24, 30, and 36 months) in breast cancer patients. The red line represents mean values at each time point; fold changes were calculated relative to the baseline. Individual patient trajectories are shown as gray lines, with the mean fold change highlighted in red. (D) Left ventricular systolic velocity (LVS′) was measured using pulsed-wave tissue Doppler imaging at the lateral mitral annulus to assess the systolic velocity of the left ventricle at TZ Hospital. (A-D) Longitudinal changes relative to baseline were analyzed using linear mixed-effects models accounting for within-patient correlation, with baseline-referenced post hoc contrasts adjusted for multiple comparisons (∗*P <* 0.05, ∗∗*P <* 0.01). (E) Fold changes of LVESV, LVEDV, LVEF, and LVS′ were stratified by treatment groups: conventional anthracyclines, liposomal doxorubicin, and anthracyclines + 5-FU. Each dot represents a patient, and shaded areas represent the 95% CI.

To evaluate the cardiotoxic effects of anthracycline-based chemotherapy, we longitudinally assessed echocardiographic parameters in 89 breast cancer patients treated with DOX or epirubicin (mean age 57.5 ± 10.3 years), including 2 male patients, with the majority of tumors classified as high grade. Follow-up measurements were collected at baseline and at 3, 6, 12, 24, 30, and 36 months post-treatment (Tables 1 and 2). M-mode echocardiography revealed a significant increase in LVESV at 12 months post-treatment compared with baseline (Figure 1A). Despite the lack of significant group-level differences at later time points, a subset of patients showed persistent elevations in LVESV fold change over time (Figure 1A). Similarly, although LVEDV did not show significant differences across time points (Figure 1B), fold-change plots suggested that a proportion of patients experienced notable increases in LVEDV following treatment (Figure 1B), indicating potential interindividual variation in ventricular remodeling. Additionally, LVEF declined significantly from baseline at multiple follow-up time points (Figure 1C). More than one-half of the patients demonstrated reduced LVEF post-treatment, with some exhibiting up to 20% declines, which is in agreement with previous studies.^26^^,^^27^ Analysis of LVEF fold changes demonstrated a consistent downward trend across the cohort (Figure 1C). On the other hand, systolic LVS′, measured by tissue Doppler imaging, did not display a consistent pattern at the group level following anthracycline treatment (Figure 1D). Although the majority of patients showed only modest changes in LVS′, generally within ±0.2-fold of baseline, a distinct subgroup demonstrated a notable increase in fold change, especially at the 3-month time point (Figure 1D), indicating considerable interindividual variability and suggesting a potential early compensatory response in myocardial contractile function. Together, these findings suggest that anthracycline therapy is accompanied by early changes in ventricular volumes in a subset of patients and reductions in LVEF in a significant proportion of the cohort, potentially contributing to adverse cardiac remodeling consistent with early-stage dilated cardiomyopathy.

**Table 1:** Summary of Clinicopathological Data of Plasma Samples From 89 Breast Cancer Patients

Age, y	57.5 ± 10.3
Histological grade^a^	
Low grade	7 (7.9)
High grade	79 (88.8)
Unknown	3 (3.4)
Estrogen receptor (+)	67 (75.3)
Progesterone receptor (+)	50 (56.2)
Epidermal growth factor receptor 2	
+	32 (36.0)
−	53 (59.6)
Unknown	4 (4.5)

Values are mean ± SD or n (%).

a Grading: Low grade, G1; high grade, G2-3.

**Table 2:** ROC Performance of m*PIH1D1* at 3 Months and Combined 3 and 6 Months

m*PIH1D1*	ROC Results
3 mo	
Sample size available	68
Positive^a^	4
Cutoff	2.974
AUC (95% CI)	0.78595% (0.505-1.000)
Sensitivity (95% CI)	0.75 (0.158-1.000)
Specificity (95% CI)	0.75 (0.636-0.859)
3 and 6 mo	
Sample size available	57
Positive	3
Cutoff	pˆ = 0.152
AUC (95% CI)	0.951 (0.864-1.000)
Sensitivity (95% CI)	1.00 (0.333-1.000)
Specificity (95% CI)	0.907 (0.815-1.000)

m*PIH1D1* = methylated PIH1 domain containing 1.

a Positive: Patients with a ≥1.5-fold increase in both left ventricular end-diastolic and end-systolic volumes at any time point were classified as positive.

### Liposomal-DOX treatment showed a more stable LVEF

To evaluate the impact of different anthracycline-based chemotherapeutic regimens on cardiac function, we compared echocardiographic measurements among patients treated with liposomal DOX, conventional anthracyclines, or anthracyclines in combination with 5-fluorouracil (5-FU) (Figure 1E). No significant changes were observed in LVESV, LVEDV, or LVS′ across all groups. In particular, patients treated with liposomal DOX exhibited consistently stable LVEF levels during the follow-up period in the fold-change plot. In contrast, patients treated with conventional anthracyclines, particularly those receiving combination therapy with 5-FU, demonstrated more pronounced cardiac alterations. Patients receiving anthracyclines in combination with 5-FU exhibited the greatest increase in LVEDV and LVESV, as well as the most substantial decline in LVEF, suggesting both chamber dilation and compromised systolic function. Although LVS′ remained relatively stable across groups.

Patients treated with conventional anthracyclines were further analyzed to evaluate the potential impact of concurrent herceptin therapy and radiotherapy on cardiac function. No significant changes in LVEDV, LVESV, LVEF, or LVS′ were observed over time, regardless of herceptin treatment status (Supplemental Figure S1) or among patients who received radiotherapy with a mean heart dose (MHD) below 1,000 cGy (Supplemental Figure S2). However, patients exposed to higher MHD levels (>1,000 cGy) demonstrated a more pronounced reduction in LVEF and an apparent increase in LVS′. Taken together, these results highlight the varying degrees of cardiotoxicity associated with different anthracycline-based treatment regimens. Liposomal DOX appears to offer relative cardioprotection, whereas the addition of 5-FU and exposure to high-dose radiotherapy may contribute to increased cardiac remodeling. Larger, well-powered studies will be necessary to validate these observations and better define the individual and combined contributions of these therapeutic components to long-term cardiac outcomes.

### Elevated hs-cTnI levels are associated with reduced ejection fraction

**Figure 2:**
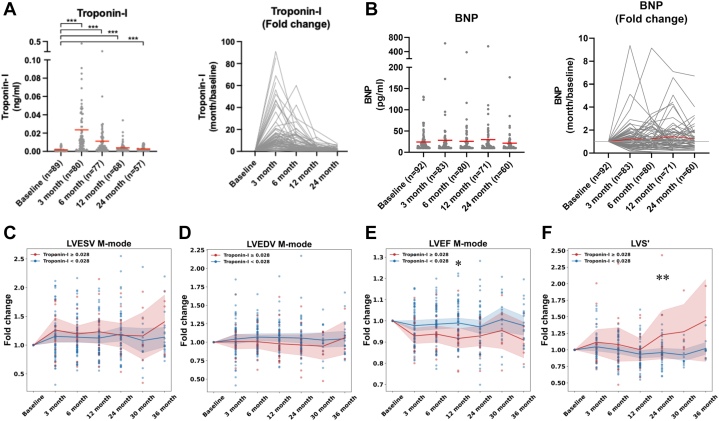
Higher Elevated Troponin-I (High-Sensitivity Cardiac Troponin I) Levels Are Associated With Reduced Ejection Fraction (A) High-sensitivity cardiac troponin I (hs-cTnI) and (B) B-type natriuretic peptide (BNP) levels were measured at baseline (pretreatment) and various post-treatment (3, 6, 12, and 24 months). The red line represents mean values at each time point; fold changes were calculated relative to the baseline. Individual patient trajectories are shown as gray lines, with the mean fold change highlighted in red. Longitudinal changes relative to baseline were analyzed using linear mixed-effects models accounting for within-patient correlation, with baseline-referenced post hoc contrasts adjusted for multiple comparisons. Fold changes of (C) LVESV, (D) LVEDV, (E) LVEF, and (F) LVS′ were relative to baseline over time points (3, 6, 12, 24, 30, and 36 months) in patients stratified by high-sensitivity cardiac troponin I levels: troponin ≥0.028 (red line) and troponin <0.028 (blue line). Each point represents an individual patient, with means shown by the lines for each group, and shaded areas represent the 95% CI. Between-group differences over time were analyzed using repeated-measures models accounting for within-patient correlation, with *P* values adjusted for multiple comparisons. Statistical significance is indicated by asterisks. Statistical significance is indicated by asterisks (∗*P <* 0.05, ∗∗*P <* 0.01, ∗∗∗*P <* 0.001). Abbreviations as in Figure 1.

We further evaluated clinically established biomarkers of cardiac injury in this cohort of breast cancer patients. Unlike echocardiographic parameters, plasma troponin-I (hs-cTnI) levels showed a significant increase within the first 3 months following treatment but gradually declined thereafter, returning close to baseline levels by 12 months (Figure 2A). In contrast, BNP levels did not show significant changes following DOX treatment in our patient group (Figure 2B). To determine whether hs-cTnI levels reflected the cardiac changes observed in our cohort, we applied a clinical cutoff of 0.028 ng/mL, a threshold commonly used for early detection of acute myocardial infarction,^28^^,^^29^ at the 3-month time point. Patients with higher hs-cTnI levels (≥0.028 ng/mL; red line) (Figures 2C and 2D) did not exhibit significant differences in LVESV and LVEDV, as compared with those with lower levels (<0.028 ng/mL; blue line) (Figures 2C and 2D). However, elevated hs-cTnI levels were associated with changes in cardiac function, showing a strong negative correlation with LVEF, while patients with higher hs-cTnI levels demonstrated a significantly greater decline in LVEF (red line) (Figure 2E). Additionally, higher hs-cTnI levels were associated with increased changes in myocardial contractility (LVS), although only one of the time points reached statistical significance (Figure 2F). In summary, elevated hs-cTnI levels appear to reflect greater impairment in cardiac function, particularly in terms of LVEF decline, but are not reliable indicators of changes in ventricular volumes. These findings underscore the limitations of current biomarkers in detecting subtle forms of anthracycline-induced cardiotoxicity, such as early systolic and diastolic dilation, and highlight the need for more sensitive and specific markers.

### Identification and validation of heart-specific methylated CpG sites in *PIH1D1*

**Figure 3:**
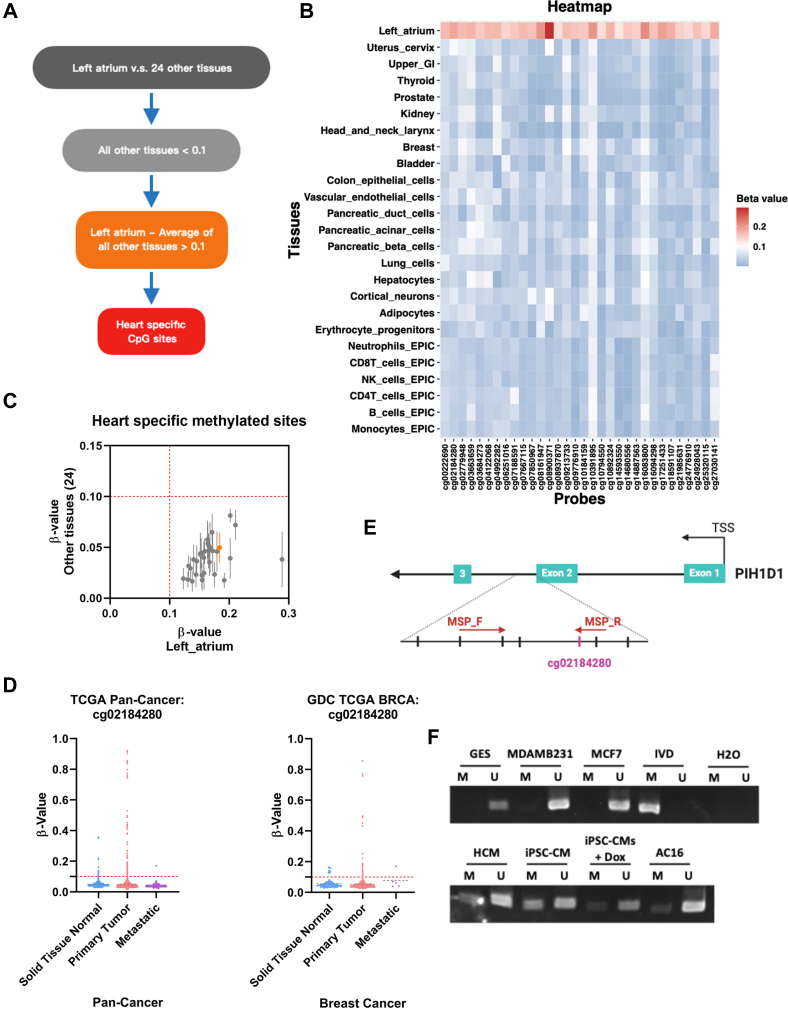
Identification and Validation of Heart-Specific Methylated CpG Sites in *PIH1D1* (A) Workflow illustrating the selection criteria for heart-specific methylated CpG sites (HSMCs). CpG sites were filtered by comparing methylation levels in the left atrium to those in 24 other tissues. Sites with methylation levels below 10% in noncardiac tissues were first selected. CpG sites showing a methylation difference >10% between the left atrium and the average of other tissues were further classified as HSMCs. (B) A total of 33 CpG sites met these criteria and were classified as HSMCs. Heatmap showing the methylation β-values of these 33 HSMCs across 25 different human tissues. (C) Scatter plot showing the β-values of the left atrium (x-axis) compared with the average β-values of 24 other tissues (y-axis) with SD error bars. The orange dot highlights *PIH1D1*, representing a CpG site of interest for further investigation. (D) DNA methylation β-values of *PIH1D1* (cg02184280) across The Cancer Genome Atlas (TCGA) and The Genomic Data Commons (GDC) data sets, including pan-cancer and breast cancer cohorts. β-values remain below 10% in primary tumors, adjacent normal tissues, and metastatic samples, with higher hypomethylation specificity observed in breast cancer patients. (E) Schematic representation highlighting the CpG site cg02184280 (purple) and the design of methylation-specific PCR (MSP) primers (red) and bisulfite pyrosequencing primers (orange). (F) Gel-based MSP shows amplicons in primary human cardiomyocytes (HCM), induced pluripotent stem cell–derived cardiomyocytes (iPSC-CMs), and AC16 cells but not in GES or breast cancer cell lines. Amplicons were also observed in doxorubicin-treated iPSC-CMs, indicating the specificity of the detected CpG sites to cardiac tissue methylation patterns. In vitro methylated human DNA (IVD) was used as a positive control to validate the specificity and efficiency of the methylation-specific primers. “M” indicates detection of methylated *PIH1D1*, while a product in the “U” lane indicates unmethylated *PIH1D1*.

We next investigated whether DNA methylation, a stable and informative biomarker in bodily fluids,^30^ could serve as an early biomarker for anthracycline-induced cardiotoxicity. We hypothesized that heart-specific methylated DNA fragments may be released into the circulation following anthracycline-mediated cardiotoxicity. To identify the HSMCs, we utilized a comprehensive human methylome reference atlas, including data from 25 different human tissues or cell types.^25^ We applied a 2-step filtering strategy (Figure 3A) (details in the Methods section), which yielded 33 candidate HSMCs (Figure 3B). Notably, a distinct cluster of elevated methylation was observed exclusively in left atrial samples, the only cardiac tissues available in that data set. Among these, *PIH1 Domain Containing 1* (*PIH1D1*) was identified as a strong candidate because of its high methylation in the left atrium and minimal signal across all other tissues (Figure 3C). We first investigated if methylation of *PIH1D1* is altered in malignancy. Data from the TCGA and GDC revealed that *PIH1D1* exhibited consistent hypomethylation levels below 10% in most cancer patients in tumor tissues as well as the adjacent normal tissues in the pan-cancer cohort. Importantly, in the breast cancer (BRCA) cohort, the hypomethylation of *PIH1D1* was even more pronounced, with only a few patients showing methylation levels higher than 10% in tumor samples. These findings highlight that *PIH1D1* remains largely unmethylated, even in the context of malignancy, particularly among breast cancer patients (Figure 3D).

To further validate the tissue specificity of *PIH1D1* methylation, methylation-specific PCR (MSP) was performed across various cell types. MSP results showed that partial methylation of *PIH1D1* was detected exclusively in primary human cardiomyocytes, iPSC-CMs, and the immortalized human cardiomyocyte line AC16, while other cell types showed no detectable methylation (Figures 3E and 3F). Treatment of iPSC-CMs with DOX did not result in substantial demethylation of *PIH1D1*, despite previous reports indicating that DOX induces global DNA hypomethylation, particularly in cardiac tissues, suggesting that *PIH1D1* methylation remains largely preserved under these conditions^31^, ^32^, ^33^, ^34^ (Figure 3F). Based on this cardiac-specific and DOX-resistant methylation pattern, these CpG regions were selected for further analysis of *PIH1D1* methylation in plasma cfDNA from breast cancer patients in our cohort.

### Elevated plasma level of m*PIH1D1* is associated with cardiac remodeling in breast cancer patients

**Figure 4:**
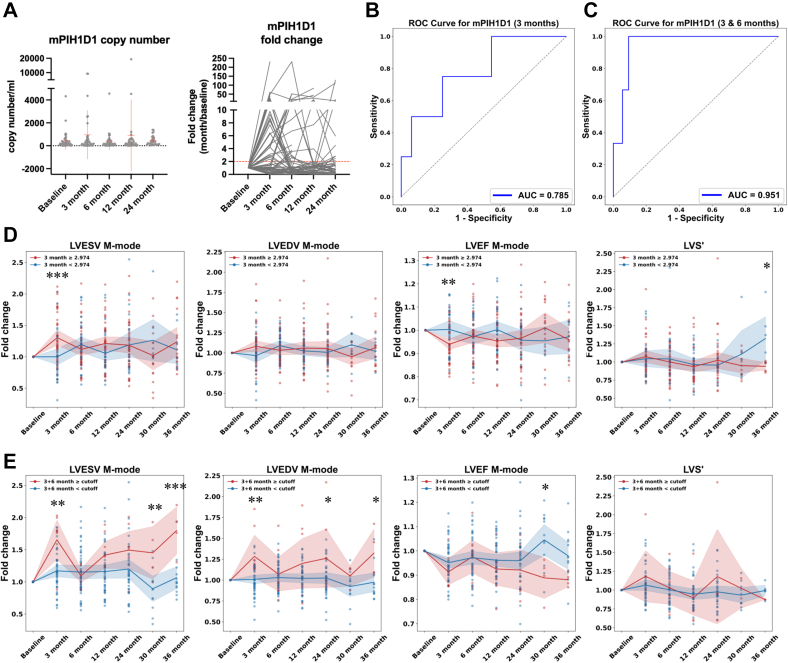
Detection of mPIH1D1 Levels in the Plasma Predicting Diastolic and Systolic Changes in Breast Cancer Patients (A) Quantification of m*PIH1D1* levels in plasma by qMSP. Scatter plot showing m*PIH1D1* copy numbers at baseline and at 3, 6, 12, and 24 months post-treatment. Each dot represents a patient sample, and red bars indicate the median with the IQR. Fold change in m*PIH1D1* levels relative to baseline across time points. (B) Receiver-operating characteristic (ROC) curve analysis of m*PIH1D1* levels for predicting ventricular dilation. Patients with a ≥1.5-fold increase in both LVEDV and LVESV at any time point were classified as positive. ROC at 3 months yielded an AUC of 0.785 (95% CI: 0.505-1.000) with both sensitivity and specificity of 0.75 (sensitivity: 95% CI: 0.158-1.000; specificity: 95% CI: 0.636-0.859), and (C) combining 3- and 6-month data yielded an AUC of 0.951 (95% CI: 0.864-1.000) with 100% (95% CI: 0.333-1.000) sensitivity and 90.7% (95% CI: 0.815-1.000) specificity. (D) Longitudinal fold changes of echocardiographic parameters (LVESV, LVEDV, LVEF, LVS′) stratified by fold change of m*PIH1D1* values at 3 months (≥2.974 vs < 2.974). (E) Similar analysis stratified by fold change of m*PIH1D1* using the combined predicted probability (pˆ) between 3- and 6-month time points, with a defined cutoff (pˆ ≥ 0.152 or pˆ < 0.152). In (D) and (E), each dot represents a patient; lines represent group means. Shaded areas indicate 95% CIs. Between-group differences over time were analyzed using repeated-measures models accounting for within-patient correlation, with *P* values adjusted for multiple comparisons. Statistical significance is indicated by asterisks (∗*P <* 0.05, ∗∗*P <* 0.01, ∗∗∗*P <* 0.001). Abbreviations as in Figure 1.

To evaluate the potential of methylated *PIH1D1* (m*PIH1D1*) as a biomarker for detecting anthracycline-induced cardiotoxicity, we quantified m*PIH1D1* levels in plasma samples from breast cancer patients over time using qMSP (Figure 4A). A subset of patients exhibited a significant increase in m*PIH1D1* copy number following anthracycline treatment, with fold changes ranging from 2-fold to as high as 200-fold. To assess the diagnostic performance of m*PIH1D1*, we conducted ROC curve analysis. Patients with both LVEDV and LVESV exceeding 1.5-fold at any follow-up time point relative to the baseline were categorized as “positive” for ventricular dilation, while all other patients were classified as “negative.” At 3 months post-treatment, the fold change in m*PIH1D1* copy number yielded an AUC of 0.785, with both sensitivity and specificity of 0.75 (Figure 4B, Table 2). When combining m*PIH1D1* fold change values from both the 3- and 6-month time points, the classification performance improved substantially, achieving an AUC of 0.951 with 100% sensitivity and 90.7% specificity (Figure 4C, Table 2), highlighting the strong potential of m*PIH1D1* fold change as an early indicator of anthracycline-induced cardiac dysfunction.

To directly compare the discriminatory value of troponin-I with m*PIH1D1*, we performed ROC analyses using troponin-I levels at 3 and 6 months to distinguish patients with ≥1.5-fold increases in both LVEDV and LVESV, following the same criteria. Troponin-I at 3 months yielded an AUC of 0.658, while the combined model at 3 and 6 months modestly improved to an AUC of 0.719 (Supplemental Figure S3). These results support the limited utility of troponin-I for detecting early structural remodeling compared with m*PIH1D1*.

We next stratified patients based on a 3-month m*PIH1D1* fold-change cutoff of 2.974 relative to baseline levels, as determined by ROC analysis. The higher m*PIH1D1* group showed greater LVESV increase and a larger reduction in LVEF at 3 months compared with the lower group (Figure 4D). However, beyond the early post-treatment period, group trajectories largely overlapped and did not show consistent separation across later follow-up time points, suggesting that 3-month m*PIH1D1* primarily captures early functional changes rather than long-term cardiac remodeling. To better capture ventricular remodeling, patients were further stratified according to their combined m*PIH1D1* fold change values at 3 and 6 months relative to baseline. Methylation of *PIH1D1* clearly distinguished patients with increased LVEDV and LVESV, as well as those with persistent declines in LVEF (Figure 4E). Although no consistent directional trend was observed in LVS′, patients with elevated m*PIH1D1* levels exhibited greater variability in myocardial velocity over time.

We also examined whether hormone receptor status or hormone therapy might influence m*PIH1D1* levels or cardiac remodeling. Progesterone receptor status had no measurable effect on m*PIH1D1* copy number or fold change at any time point (Supplemental Figure S4B). In contrast, Estrogen receptor (ER)-negative patients exhibited slightly lower m*PIH1D1* copy numbers, especially at 12 months, although their longitudinal fold changes were comparable to those of ER-positive patients (Supplemental Figure S4A). These findings suggest that ER status may influence the basal level of circulating m*PIH1D1* after treatment, but not its temporal dynamics, indicating that ER expression is unlikely to affect the utility of m*PIH1D1* for early detection. Furthermore, patients treated with Femara displayed a trend toward greater LVESV dilation and a more pronounced LVEF decline compared with those receiving Tamoxifen or no hormone therapy (Supplemental Figure S5). Although exploratory, these findings suggest possible hormone-related modulation of cardiac remodeling and warrant further investigation.

In summary, plasma m*PIH1D1*-fold change, particularly the combined 3+6-month metric, effectively identified patients who subsequently developed ≥1.5-fold increases in LVEDV/LVESV (AUC 0.951; sensitivity 100%; specificity 90.7%). Together, these findings support m*PIH1D1* as a heart-derived cfDNA methylation marker of early ventricular remodeling and highlight the need for prospective validation as a biomarker.

## Discussion

Long-term monitoring of anthracycline-treated cancer patients is essential because of the risk of cardiotoxicity, which may manifest years after treatment. In this study, we present a comprehensive follow-up study of breast cancer patients treated with anthracyclines, integrating echocardiographic evaluations and biomarker analyses. These findings further emphasize the critical need for continuous cardiac surveillance in this patient population. Previous studies have established a strong association between anthracycline treatment and a subsequent decline in LVEF, a key indicator of cardiotoxicity that can progress to heart failure.^35^, ^36^, ^37^ Consistent with these findings, over 50% of patients in our cohort exhibited a reduction in LVEF. In addition to this functional impairment, we observed significant time-dependent left ventricular dilation during both diastolic and systolic phases, as evidenced by increased LVEDV and LVESV. These changes are indicative of progressive ventricular remodeling. Such structural remodeling is often a compensatory response aimed at maintaining stroke volume in the setting of reduced ejection fraction and diastolic dysfunction. This pattern aligns with the hallmark features of dilated cardiomyopathy and highlights the pivotal role of anthracycline treatment in the development of heart failure and related cardiac pathologies.^38^ Notably, in our cohort, these dilated changes persisted for at least up to 2 years post-treatment, highlighting the prolonged and progressive effects of anthracyclines on cardiac structure and function.

Consistent with prior reports,^39^^,^^40^ liposomal DOX was associated with more favorable cardiac trajectories than conventional anthracyclines. In our cohort, LVEF in the liposomal group remained stable over follow-up, whereas patients receiving conventional anthracyclines showed a progressive decline. Likewise, LVEDV and LVESV in the liposomal group were stable or slightly decreased, contrasting with the modest dilation observed with conventional regimens. Conversely, the pronounced decline in LVEF observed in patients receiving 5-FU–containing regimens highlights the additive cardiotoxic risk posed by this combination. This finding aligns with previous reports indicating that 5-FU exacerbates myocardial damage when combined with anthracyclines, emphasizing the importance of careful consideration and monitoring when prescribing this regimen in clinical practice.^41^^,^^42^

Subgroup analyses further evaluated the impact of herceptin and radiotherapy on cardiac function within the conventional anthracycline cohort. Consistent with previous studies, our data indicate that herceptin does exhibit cardiotoxic effects, as evidenced by a trend toward lower LVEF in treated patients.^43^ However, this effect may not be the primary contributor to cardiotoxicity in this regimen, because the changes were not statistically significant. Regarding radiotherapy, a greater decline in LVEF was observed in patients with an MHD exceeding 1,000. However, the number of patients reaching this cutoff was limited, reducing the statistical confidence in this observation. Conversely, when analyzing data with MHD cutoffs below 1,000, no significant differences in LVEF were observed. These findings suggest that maintaining MHD below 1,000 may be relatively safe for patients undergoing this regimen combination, although further studies with larger sample sizes are needed to validate these thresholds.

Cardiac troponin-I, a well-established biomarker for cardiac injury, has been extensively studied in the context of anthracycline-induced cardiotoxicity. Although several studies have reported that elevations in troponin-I can precede declines in LVEF, other reports highlight its limited sensitivity in certain patient populations and time points, underscoring the variability in its predictive value.^44^ In our cohort, elevated troponin-I levels were observed during the early post-treatment phase, consistent with previous studies identifying its transient elevation as an indicator of acute cardiac damage.^45^^,^^46^ However, these levels gradually returned to baseline by 12 months, illustrating its limitations in reflecting long-term structural changes. In similar studies, elevated troponin-I levels can serve as an early warning signal for functional impairments, such as a decline in LVEF.^47^^,^^48^ However, it appears less effective in capturing progressive remodeling processes like ventricular dilation. These results highlight the need for complementary biomarkers capable of providing a more comprehensive evaluation of anthracycline-induced cardiotoxicity, including subtle yet clinically significant structural changes.

We, therefore, propose that detecting HSMCs could complement currently available markers by enabling early detection of minor cardiac damage and structural changes. Our findings demonstrate that fold changes of m*PIH1D1* values are strongly associated with ventricular dilation during both diastolic and systolic phases, achieving an AUC of 0.95, with 100% sensitivity and 90.7% specificity at the optimal cutoff. Nearly all cases of pronounced ventricular dilation (eg, LVEDV and LVESV both >1.5-fold) were observed in patients with increased m*PIH1D1* levels, reinforcing its utility in early detection of anthracycline-induced structural cardiac changes.

To further enhance the performance of this approach, future studies could explore additional HSMCs and evaluate their combined utility with m*PIH1D1*. A panel of HSMCs might improve sensitivity and specificity, enabling a more comprehensive assessment of anthracycline-induced cardiotoxicity. Additionally, integrating m*PIH1D1* or other HSMCs into biomarker workflows using single-molecule sequencing could provide a rapid, cost-effective, and high-throughput solution for clinical applications. This approach has the potential to streamline the detection process, making it more accessible for real-time monitoring and personalized risk assessment in patients undergoing anthracycline-based chemotherapy. Future prospective trials integrating m*PIH1D1* into cardiotoxicity surveillance protocols and testing its responsiveness to cardioprotective therapies (eg, beta-blockers, dexrazoxane) will be essential to establish its clinical utility.

### Study limitations

First, although the 89 breast cancer patients were followed prospectively over 3 years, the sample size was modest and limited the power of subgroup analyses. Larger and long-term, multicenter cohorts are needed to validate m*PIH1D1* across diverse populations and treatment settings, particularly in relation to sustained, non-reversible LV remodeling. Second, the cohort was restricted to breast cancer patients, which may limit generalizability. Broader evaluation in other cancers, such as lymphoma and sarcoma, and under varying treatment regimens, is warranted. Third, although M-mode echocardiography is practical and reproducible, it lacks the sensitivity of more advanced imaging modalities. It was chosen to minimize patient burden during postsurgical recovery and chemotherapy. Future studies incorporating advanced imaging modalities such as 3-dimensional echocardiography, global longitudinal strain, or cardiac magnetic resonance imaging may improve the detection of subclinical myocardial injury and provide deeper insights into the temporal relationship between structural changes and biomarker dynamics. Finally, while qMSP offers high specificity, it lacks the resolution to detect low-abundance or fragment-level methylation changes. In contrast, nanopore sequencing provides broader methylome coverage and real-time methylation detection without the need for bisulfite conversion. Its portability and rapid turnaround further support its potential for point-of-care applications and future biomarker discovery.

## Conclusions

m*PIH1D1* has the potential to serve as a noninvasive biomarker for anthracycline-induced cardiac remodeling, complementing existing markers like troponin-I. Its integration into biomarker panels or high-throughput workflows could enhance personalized risk assessment and enable timely interventions to manage cardiotoxicity in breast cancer patients undergoing anthracycline-based chemotherapy.

### Data Availability Statement

All data are available in this study.

Perspectives **COMPETENCY IN MEDICAL KNOWLEDGE:** Anthracycline-based chemotherapy is associated with a substantial risk of delayed cardiotoxicity, characterized by progressive ventricular remodeling and eventual heart failure. Using circulating cell-free DNA methylation profiling, this study identifies elevated methylation of *PIH1D1* at 3 and 6 months as an early biomarker for subsequent ventricular dilation and a decline in left ventricular ejection fraction in patients with breast cancer, preceding conventional injury markers such as troponin-I. These findings highlight the potential of cfDNA methylation analysis to detect subclinical myocardial remodeling before overt functional impairment, supporting its integration into longitudinal surveillance strategies to guide timely cardioprotective intervention.

**TRANSLATIONAL OUTLOOK:** Current clinical surveillance for anthracycline-induced cardiotoxicity relies primarily on serial cardiac imaging and conventional circulating biomarkers. This study demonstrates that methylation of *PIH1D1* in circulating cfDNA serves as an early biomarker of anthracycline-associated cardiac remodeling in patients with breast cancer. Integration of m*PIH1D1* with established biomarkers, such as troponin-I, may improve the sensitivity of current monitoring strategies, enabling earlier identification of at-risk patients and facilitating timely initiation of cardioprotective therapy, or adjustment of chemotherapy regimens. Further validation of m*PIH1D1* in larger, multicenter cohorts will be essential to establish its robustness and generalizability across diverse patient populations and treatment protocols.

## Funding Support and Author Disclosures

This study was supported by grants from the European ERA-NET, ERA-CVD-JC2016, French government managed by Agence Nationale de la Recherche (ANR-16-ECVD-0005-01) Centre National de la Recherche Scientifique, Université de Strasbourg, National Science and Technology Council, Taiwan (NSTC 113-2314-B-194-002-MY3, 111-2923-B-194-001-MY3, 110-2914-B-194-002-MY3, 106-2923-B-194-001-MY3), and the Research Center for Precision Environmental Medicine, Kaohsiung Medical University, Kaohsiung, Taiwan from The Featured Areas Research Center Program within the framework of the Higher Education Sprout Project by the Ministry of Education (MOE) in Taiwan and by Kaohsiung Medical University Research Center Grant (KMU-TC113A01) to Dr Chan. All other authors have reported that they have no relationships relevant to the contents of this paper to disclose.
