# Supplementary material for: Methylated PIH1D1 as a Heart-Specific Biomarker for Anthracycline-Induced Cardiac Remodeling in Breast Cancer Patients
Source: JACC Basic Transl Sci. 2026 Mar 11;11(4):101510. doi: 10.1016/j.jacbts.2026.101510 (PMC12995573; doi:10.1016/j.jacbts.2026.101510)
Supplement: Supplementary Figures 1-5 and Supplementary Table 1 [file mmc1.docx]

**Supplemental Appendix**

**Supplementary Figure 1.**

**Supplementary Figure S1. Echocardiographic changes in patients treated with conventional anthracyclines with and without Herceptin treatment.**

Time-dependent changes in fold changes of echocardiographic parameters shown, including LVESV, LVEDV, LVS’, and LVEF, are presented for patients receiving conventional anthracycline chemotherapy. Patients were stratified into two groups based on whether they received Herceptin treatment (Herceptin: red, No Herceptin: blue). Fold changes were calculated relative to baseline measurements. Each plot shows individual patient data points, group mean values connected over time points (Baseline, 3, 6, 12, 24, 30, and 36 months), and shaded areas indicate 95% confidence intervals (CI). The data illustrate the potential impact of Herceptin on cardiac structure and function over time in this patient cohort.

**Supplementary Figure 2.**

**Supplementary Figure S2. Echocardiographic changes in patients treated with conventional anthracyclines stratified by MHD cutoff values.**

Time-dependent fold changes in echocardiographic parameters, including LVESV, LVEDV, LVS’, and LVEF, are shown for patients who received conventional anthracycline chemotherapy. Patients were stratified into groups based on MHD (mean heart dose) cutoff values: MHD >= 500, 800, 100 (red), MHD < 500, 800, 1000 (blue), and MHD = 0 (yellow). Fold changes were calculated relative to baseline measurements. Each plot displays individual patient data points and group mean values connected over time points (Baseline, 3, 6, 12, 24, 30, and 36 months). Shaded areas indicate 95% confidence intervals (CI). These data highlight the effects of mean heart dose on cardiac remodeling and function during long-term follow-up.

**Supplementary Figure 3.**

**B**

**A**

**Supplementary Figure S3. ROC curves for troponin-I in predicting anthracycline-induced ventricular remodeling.**

(A) ROC curve using troponin-I levels at 3 months to predict >= 1.5-fold increases in both LVEDV and LVESV. The area under the curve (AUC) was 0.427, with an optimal cutoff (Youden index) of 0.078, yielding 50.0% sensitivity and 64.6% specificity. (B) ROC curve based on logistic regression combining troponin-I levels at 3 and 6 months. The model achieved an AUC of 0.489, with an optimal cutoff of 0.072, 66.7% sensitivity, and 58.1% specificity. Compared to mPIH1D1 (AUC = 0.951), these results suggest limited predictive utility of troponin-I for early detection of structural remodeling.

**Supplementary Figure 4.**

**C**

**D**

**B**

**A**

**Supplementary Figure S4. mPIH1D1 copy number and fold change stratified by estrogen receptor (ER) and progesterone receptor (PR) status.**

(A) mPIH1D1 copy number (log scale) in ER+ and ER− patients across time points (baseline, 3, 6, 12, and 24 months). (B) mPIH1D1 copy number in PR+ and PR− patients. (C) log2-transformed fold change of mPIH1D1 in ER+ and ER− patients. (D) log2-transformed fold change of mPIH1D1 in PR+ and PR− patients. P values are from Mann–Whitney U tests.

**
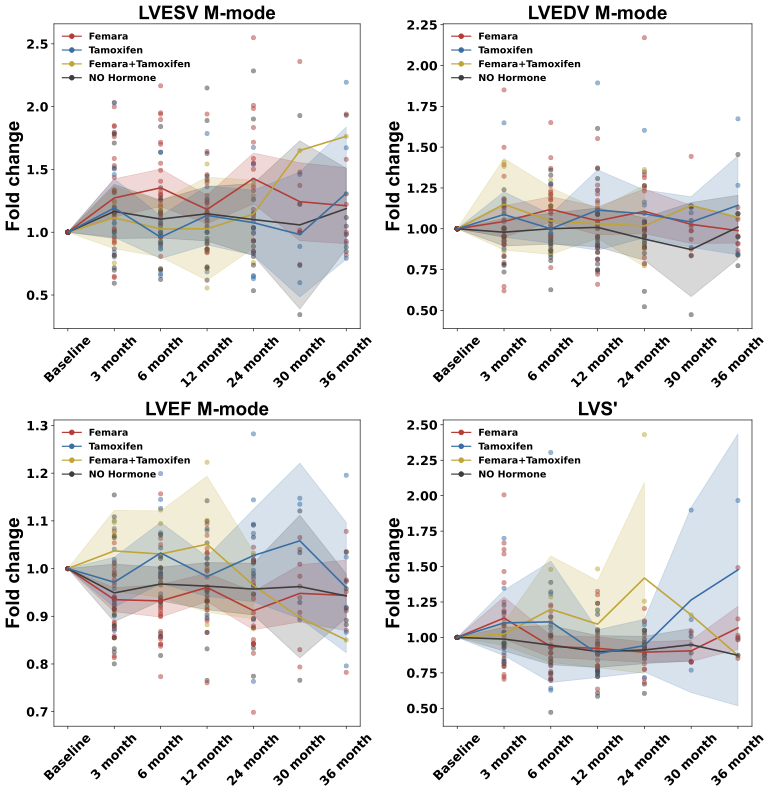
Supplementary Figure 5.**

**Supplementary Figure S5. mPIH1D1 copy number and fold change stratified by estrogen receptor (ER) and progesterone receptor (PR) status.**

Longitudinal fold change in LVESV, LVEDV, LVEF, and LVS’ measured by M-mode echocardiography from baseline to 36 months, stratified by hormone therapy group (Femara, Tamoxifen, Femara + Tamoxifen, or no hormone therapy). Each line represents the group mean, with shaded areas indicating the 95% confidence interval.

**Supplementary Table. 1 Primer list**

| Primers | Primer sequence (5’ to 3’) |
| --- | --- |
| Bisulfite pyrosequencing | |
| Pyro_PIH1D1_F | GTAGTGATAGGATTTTTGGATTTTTGAGTA |
| Pyro_PIH1D1_R_UB07 | gaacgccagcacatggacagcTTTCTTAACAAACTTAAAACCCATTAACCT |
| Pyro_PIH1D1_S | GGATTTTTGGATTTTTGAGTAT |
| MSP | |
| PIH1D1_MSP_mF | GGATTTTTGGATTTTTGAGTATTC |
| PIH1D1_MSP_mR_2 | CGAAACCTAATTCCCGC |
| PIH1D1_MSP_uF | GGATTTTTGGATTTTTGAGTATTT |
| PIH1D1_MSP_uR_2 | CCCAAAACCTAATTCCCAC |
